# Association between 4-year all-cause mortality and carnitine profile in maintenance hemodialysis patients

**DOI:** 10.1371/journal.pone.0201591

**Published:** 2018-08-22

**Authors:** Yuiko Kamei, Daigo Kamei, Ken Tsuchiya, Michio Mineshima, Kosaku Nitta

**Affiliations:** 1 Departments of Medicine, Kidney Center, Tokyo Women’s Medical University, Shinjuku, Tokyo, Japan; 2 Departments of Blood Purification, Kidney Center, Tokyo Women’s Medical University, Tokyo, Japan; 3 Departments of Clinical Engineering Tokyo Women’s Medical University, Tokyo, Japan; International University of Health and Welfare, School of Medicine, JAPAN

## Abstract

**Background:**

Patients on dialysis are in a chronic carnitine-deficient state. This condition may be associated with abnormalities of the fatty acid and organic acid metabolisms. Carnitine is required for β-oxidation of the long-chain fatty acids; therefore, carnitine deficiency decreases the efficiency of ATP synthesis and may incur death. However, the details of this association remain unknown. We examined the relationship between β-oxidation efficiency represented by the carnitine profile and 4-year all-cause mortality in hemodialysis patients.

**Methods:**

The carnitine profiles of 122 hemodialysis patients were determined by liquid chromatography-tandem mass spectrometry (LC-MS/MS). The associations between the 4-year all-cause mortality and carnitine profile as well as the clinical backgrounds of the patients were investigated. A survival analysis was conducted by the Kaplan–Meier survival method and multivariable Cox proportional hazard analysis. The bootstrap method was performed to confirm the stability and robustness of our model.

**Results:**

Of the 122 subjects analyzed, 111 were selected and 24 died during the observation period. Stepwise multivariable Cox regression demonstrated that diabetes state [Hazard ratio (95% confidence interval), 4.981 (2.107–11.77)], age [HR (95% CI), 1.052 (1.014–1.091)], and the acetylcarnitine/(palmitoylcarnitine+octadecenoylcarnitine) [C2/(C16+C18:1)] ratio [HR (95% CI), 0.937 (0.904–0.971)] were independent significant factors of 4-year all-cause mortality. The bootstrap method confirmed the significance of these three factors.

**Conclusion:**

The 4-year all-cause mortality negatively correlated with the C2/(C16+C18:1) ratio. Improvement of the impaired β-oxidation state after L-carnitine administration may ameliorate prognosis.

## Introduction

Carnitine deficiency occurs in aberrations of carnitine regulation in disorders such as diabetes, sepsis, cardiomyopathy, malnutrition, cirrhosis, endocrine disorders and with aging [1]. In hemodialysis patients, carnitine is constantly removed during the dialysis through dietary restrictions and decreased kidney function, which results in carnitine deficiency and reduced L-carnitine biosynthesis [2]. L-carnitine transports long-chain fatty acids, which are absorbed into the cytoplasm from the blood, into the mitochondria, adjusts the acyl-CoA/CoA ratio in the mitochondria, and eliminates harmful acyl-CoA. Acyl-CoA accumulates in the mitochondria of patients with abnormal organic acid and fatty acid metabolism; therefore, L-carnitine plays an important role in these metabolisms [3]. The determination of the carnitine profile can help estimate the organic acid and fatty acid metabolism dynamics of acylcarnitine in cells.

In recent years, some correlations have been reported between acylcarnitine and chronic kidney disease (CKD) exacerbation [4], anemia [5,6], and reduced physical function [7]. Carnitine deficiency in hemodialysis patients can impair the efficiency of adenosine triphosphate (ATP) synthesis from long-chain fatty acids in the mitochondria. ATP is essential for life-sustaining activities, and a decrease in the efficiency of its synthesis may be related to disease prognosis.

Here, we established the hypothesis that impaired long-chain fatty acid metabolism in chronic hemodialysis patients with L-carnitine deficiency is related to the clinical outcomes and investigated whether a correlation exists between 4-year all-cause mortality rate and the carnitine profile of the patient.

## Subjects and methods

### Subjects

We investigated 122 patients undergoing maintenance dialysis three times per week at our hospital. We excluded patients who were receiving L-carnitine, were using antibiotics with a pivoxil group for acute inflammation, and had undergone kidney transplantation or had received blood transfusion in the past 6 months.

All patients were treated in accordance with the clinical guidelines of the Japanese Society for Dialysis Therapy (JSDT) [8–13].

Written informed consent was obtained from each of the study subjects. The protocol of this study was approved by the ethics committee of Tokyo Women’s Medical University (No. 3173) and was conducted in accordance with the Helsinki Declaration of 1975 (as revised in 2000).

### Mass spectrometry measurements

Serum samples were separated immediately after blood collection in a refrigerated centrifuge, stored at −80°C until analysis, processed by the non-derivatization method, and analyzed via tandem mass spectrometry (TQD; Waters Corp., Milford, MA, USA) to determine the moieties of acylcarnitines.

We measured free carnitine: C0, acetylcarnitine: C2, propionylcarnitine: C3, isobutyrylcarnitine: C4, isovalerylcarnitine: C5, 3-hydroxyisovalerylcarnitine: C5-OH, tiglylcarnitine: C5: 1,glutarylcarnitine: C5DC, hexanoylcarnitine: C6, octanoylcarnitine: C8, decanoylcarnitine: C10, decenoylcarnitine: C10: 1,dodecanoylcarnitine: C12, tetradecanoylcarnitine: C14, tetradecenoylcarnitine: C14: 1,palmitoylcarnitine: C16, 3-hydroxypalmitoylcarnitine: C16OH, stearoylcarnitine: C18, 3-hydroxyoctadecenoylcarnitine: C18: 1-OH, and octadecenoylcarnitine: C18:1.

We also calculated six acylcarnitine ratios typically included in newborn screening [14], namely C2/C3, C8/C10, C2/C10, C2/C14:1, C0/(C16+C18), and C2/(C16+C18:1).

Very-long-chain acylcarnitines (chain length > 18) were not analyzed in this study. We measured each variable twice, and used the average value of the two measurements for calculations.

We defined short-chain acylcarnitine as the species of all acylcarnitine from C2 to C5, middle-chain acylcarnitine as the species of all acylcarnitine from C6 to C12, and long-chain acylcarnitine as the species of all acylcarnitine from C14 to C18.

### Data collection

Age, sex, dry weight, etiology of end-stage renal disease (ESRD), dialysis vintage, comorbidities, pre-dialysis blood pressure, human recombinant erythropoietin (ESA) dose, and cause of death were obtained from the patients’ medical records. Body mass index (BMI) was calculated as an individual’s dry weight (kg) divided by the square of the height (m) at study entry. The conventional urea kinetic measure, known as Kt/V (single pool), was used to estimate the dialysis dose. Blood sampling was performed in November 2013, and the observation period ended in November 2017.

### Statistical methods

Data are presented as mean ± standard deviation (SD) and as medians and interquartile range (IQR). Fisher’s exact test was used to compare sex and diabetes states between the two groups. The Mann–Whitney U test was used to compare clinical findings between the two groups.

Cox regression analysis was used to identify factors associated with all-cause mortality, with explanatory variables for age; gender; dialysis vintage; dummy variable diabetes states (yes = 1 and no = 0); body mass index (BMI); erythropoietin resistance index (ERI); and the spKt/V, β2MG, Total protein, serum albumin, CRP, whole PTH, TSAT, ferritin, and carnitine profiles. The stepwise criteria were based on the standard specifications of the SPSS software, i.e., P ≤ 0.05 for inclusion and P > 0.10 for exclusion.

We performed log-rank test and Breslow test on the Kaplan–Meier estimates on the data of patients belonging to the two groups based on the median value of carnitine profile selected by multivariable Cox regression.

According to the ASA’s statement on P values [15], we used the SPSS biased-corrected and accelerated bootstrap method with 2000 bootstrap samples and 95% confidence interval to confirm the stability and robustness of our model. P < 0.05 was considered statistically significant. All statistical analyses were performed with SPSS version 25.0 (IBM Corp.; Armonk, NY, USA).

## Results

Of the 122 patients who were undergoing maintenance hemodialysis three times per week at our hospital, 6 patients were receiving L-carnitine, 2 were taking antibiotics of the pivoxil group for acute inflammation, and 3 had undergone kidney transplantation. None of the 122 patients had a history of transfusion within the previous 6 months. Therefore, finally, 111 patients were selected for the study.

The clinical characteristics of each of the selected 111 patients are shown in Table 1 and the pre-dialysis carnitine profile is shown in Table 2.

**Table 1:** Baseline characteristics of enrolled patients (n = 111).

Variable	Mean±SD and Median [IQR]	N
**Age (year)**	59 ±14, 60[50, 69]	
**Gender (Male/Female)**		70/41
**Etiology of ESRD**		
**Glomerulonephritis**		68
**Type 2 diabetes mellitus (DM)**		21
**Nephrosclerosis**		8
**Hereditary disease**		6
**Rejection after kidney transplantation**		4
**the others**		4
**Dialysis vintage (year)**	12 ± 11, 8 [2, 21]	
**Dry weight (kg)**	54.0 ± 12.8, 54.0 [45.2, 60.0]	
**Body Mass Index (kg/m2)**	20.5±4.1, 19.8 [17.6, 22.0]	
**Hemoglobin (g/dL)**	11.0 ± 1.0, 11.0[10.4, 11.8]	
**Total protein (g/dL)**	6.7 ± 0.4, 6.7 [6.4, 7.0]	
**Albumin (g/dL)**	3.7 ± 0.4, 3.7 [3.5, 3.9]	
**BUN (mg/dL)**	62.4 ± 15.4, 63.1 [53.3, 73.5]	
**Creatinine (mg/dL)**	10.8 ±2.5, 10.8 [9.5, 12.6]	
**Uremic acid (mg/dL)**	7.6 ± 1.2, 7.6 [6.8, 8.3]	
**Potassium (mEq/L)**	4.9 ± 0.8, 5.0 [4.4, 5.4]	
**Total Calcium (mg/dL)**	8.9 ± 0.8, 8.8 [8.4, 9.4]	
**Phosphate (mg/dL)**	5.3 ± 1.3, 5.3 [4.5, 5.9]	
**Magnesium (mg/dL)**	2.0 ± 0.3, 2.0 [1.9, 2.2]	
**Transferrin saturation: TSAT (%)**	20.8 ± 10.1, 19.4 [12.7, 28.1]	
**Ferritin (ng/mL)**	57.6 ± 57.8, 36 [20, 86]	
**C-reactive protein (CRP) (mg/dL)**	0.48 ± 1.01, 0.11 [0.06, 0.31]	
**spKt/V un**	1.50 ± 0.31, 1.46 [1.29, 1.67]	
**Whole parathyroid hormone(wPTH) (pg/mL)**	132 ± 115, 105 [51, 174]	
**β2-microglobulin (mg/L)**	28.0 ± 6.0, 28.0 [24.4, 32.4]	
**Erythropoiesis-stimulating agent (ESA) doses/kg/(g/dL)/week**	11.8 ± 12.5, 9.3 [4.1, 14.1]	
**Pre-dialysis systolic blood pressure (mmHg)**	142 ± 23, 142 [127, 155]	
**Pre-dialysis diastolic blood pressure (mmHg)**	78 ± 16, 79 [69,86]	

**Table 2:** Value of each carnitine moiety (n = 111).

	Value (Mean±SD and Median [IQR])
**C0 (nmol/mL)**	22.75±8.84, 20.75 [17.03, 26.96]
**C2 (nmol/mL)**	9.079±4.057, 8.439 [5.990, 11.341
**C3 (nmol/mL)**	0.373±0.173, 0.332 [0.249, 0.449]
**C4 (nmol/mL)**	0.568±0.288, 0.506 [0.365, 0.677]
**C5 (nmol/mL)**	0.281±0.207, 0.250 [0.214, 0.294]
**C5OH (nmol/mL)**	0.187±0.065, 0.183 [0.158, 0.208]
**C5:1 (nmol/mL)**	0.054±0.020, 0.052 [0.039, 0.062]
**C5DC (nmol/mL)**	1.082±0.429, 1.025 [0.787, 1.329]
**C6 (nmol/mL)**	0.046±0.034, 0.039 [0.029, 0.053]
**C8 (nmol/mL)**	0.220±0.225, 0.178 [0.127, 0.275]
**C10 (nmol/mL)**	0.395±0.326, 0.324 [0.222, 0.502]
**C10:1 (nmol/mL)**	0.299±0.152, 0.278 [0.191, 0.378]
**C12 (nmol/mL)**	0.095±0.052, 0.084 [0.055, 0.127]
**C14 (nmol/mL)**	0.035±0.013, 0.032 [0.026, 0.043]
**C14:1 (nmol/mL)**	0.096±0.069, 0.077 [0.048, 0.128]
**C16 (nmol/mL)**	0.093±0.030, 0.090 [0.069, 0.111]
**C16 OH (nmol/mL)**	0.005±0.001, 0.005 [0.0045, 0.0060]
**C18 (nmol/mL)**	0.038±0.014, 0.035 [0.028, 0.044]
**C18:1OH (nmol/mL)**	0.008±0.003, 0.008 [0.006, 0.010]
**C18:1 (nmol/mL)**	0.128±0.055, 0.117 [0.085, 0.161]
**C2/C3**	26.7±12.8, 23.6 [19.2, 30.2]
**C8/C10**	0.561±0.112, 0.557 [0.496, 0.601]
**C2/C10**	28.0±13.5, 25.2 [18.3, 34.9]
**C2/C14:1**	125±74, 105 [74.6, 156]
**C0/(C16+C18)**	188±90, 175 [125, 221]
**C2/(C16+C18:1)**	42.0±15.2, 41.6 [32.3, 50.1]

footnote: C0, free carnitine; C2,Acetylcarnitine; C3, Propionylcarnitine; C4, Isobutyrylcarnitine; C5, Isovalerylcarnitine; C5-OH, 3-hydroxyisovalerylcarnitine; C5:1, Tiglylcarnitine; C5DC, Glutarylcarnitine; C6, Hexanoylcarnitine; C8, Octanoylcarnitine; C10, Decanoylcarnitin; C10:1, Decenoylcarnitine; C12, Dodecanoylcarnitine; C14, Tetradecanoylcarnitine; C14:1, Tetradecenoylcarnitine; C16, Palmitoylcarnitine; C16OH, 3-hydroxypalmitoylcarnitine; C18, stearoylcarnitine; C18:1 OH, 3-hydroxyoctadecenoylcarnitine; C18:1 Octadecenoylcarnitine.

The mean age of the study population was 59 ± 14 years, and 63% of the patients were men. No patient had any inborn error of organic acid metabolism or an inborn error of fatty acid metabolism.

During the 4-year period, 24 of the 111 people died, of which 8 died due to septicemia, 6 due to cardiovascular disease, 5 due to pneumonia, 3 due to cerebrovascular disease, 1 due to malignant tumor, and another 1 due to multiple organ failure.

Table 3 shows the results of the univariable Cox regression analysis between mortality and clinical background and pre-dialysis carnitine profile and bootstrap results.

**Table 3:** Univariable Cox regression analysis of the correlates of mortality, baseline characteristics, carnitine profile, and bootstrap results.

	Univariable Cox regression analysis			Bootstrap Result (2000 Replicas)			
	Hazard ratio: exp (β)	95%CI	*P*	Bias	S.E	95% CI	*P*
**Age**	1.071	1.035 to 1.109	<0.001	0.001	0.022	0.031 to 0.118	0.001
**Gender**	0.966	0.423 to 2.207	0.934	0.028	0.446	-0.881 to 0.978	0.934
**HD vintage**	1.015	0.981 to 1.050	0.385	-0.001	0.018	- 0.025 to 0.050	0.386
**diabetes states (yes = 1, no = 0)**	4.425	1.978 to 9.901	<0.001	0.004	0.434	0.663 to 2.307	<0.001
**BMI**	0.602	0.065 to 5.550	0.654	- 0.126	1.053	- 2.677 to 1.100	0.610
**ERI**	1.017	0.991 to 1.044	0.208	0.000	0.016	-0.016 to 0.048	0.221
**Hb**	0.747	0.509 to 1.095	0.135	-0.002	0.205	-0.706 to 0.094	0.130
**Total protein**	1.191	0.469 to 3.022	0.713	-0.026	0.526	-0.890 to 1.138	0.718
**Albumin**	0.571	0.191 to 1.713	0.318	0.000	0.512	-1.650 to 0.459	0.250
**BUN**	0.977	0.955 to 1.000	0.047	-0.001	0.013	-0.048 to -0.004	0.044
**Creatinine**	0.759	0.645 to 0.892	0.001	-0.009	0.083	-0.451 to -0.136	0.001
**Uremic acid**	0.566	0.397 to 0.809	0.002	-0.017	0.225	-1.016 to -0.163	0.006
**Potassium**	0.749	0.440 to 1.274	0.287	-0.008	0.320	-0.942 to 0.315	0.351
**Total Calcium**	1.186	0.716 to 1.966	0.507	0.003	0.259	-0.334 to 0.731	0.460
**Phosphate**	0.812	0.570 to 1.155	0.247	-0.013	0.181	-0.571 to 0.099	0.244
**Magnesium**	0.386	0.104 to 1.438	0.156	-0.019	0.616	-2.286 to 0.164	0.100
**Transferrin saturation**	0.002	0.000 to 0.203	0.009	-0.147	2.057	-11.110 to -2.829	0.002
**Ferritin**	0.997	0.988 to 1.005	0.440	-0.001	0.004	-0.012 to 0.002	0.310
**CRP**	1.169	0.875 to 1.561	0.291	0.012	0.190	-0.237 to 0.553	0.270
**spKt/V un**	1.114	0.312 to 3.975	0.868	-0.034	0.654	-1.210 to 1.252	0.860
**whole PTH**	0.994	0.989 to 1.000	0.048	0.000	0.003	-0.011 to -0.002	0.014
**β2-microglobulin**	0.995	0.932 to 1.062	0.882	0.000	0.032	-0.073 to 0.056	0.880
**Pre-dialysis systolic blood pressure**	1.010	0.993 to 1.027	0.263	0.000	0.009	-0.008 to 0.026	0.243
**Pre-dialysis diastolic blood pressure**	0.996	0.973 to 1.019	0.737	-0.001	0.013	-0.029 to 0.020	0.730
**C0**	0.896	0.831 to 0.965	0.004	-0.009	0.053	-0.229 to -0.037	0.025
**C2**	0.802	0.694 to 0.927	0.003	-0.013	0.088	-0.416 to -0.091	0.005
**C3**	0.205	0.013 to 3.123	0.254	-0.340	1.858	-5.580 to 0.736	0.327
**C4**	0.247	0.043 to 1.413	0.116	-0.180	1.033	-3.526 to -0.025	0.138
**C5**	0.000	0.000 to 0.008	0.001	-0.474	4.739	-22.620 to -5.376	0.007
**C5OH**	0.000	0.000 to 0.003	0.001	-0.410	6.223	-27.550 to -4.669	0.018
**C5:1**	0.000	0.000 to 0.000	0.008	-1.406	12.377	-62.904 to -15.866	0.004
**C5DC**	0.316	0.107 to 0.932	0.037	-0.043	0.499	-2.237 to -0.342	0.012
**C6**	0.000	0.000 to 40618	0.308	-2.946	14.134	-45.173 to 5.294	0.367
**C8**	0.093	0.001 to 7.674	0.292	-0.492	2.423	-8.339 to 0.585	0.326
**C10**	0.504	0.076 to 3.366	0.480	-0.152	0.979	-2.923 to 0.881	0.430
**C10:1**	0.113	0.005 to 2.719	0.179	-0.102	1.279	-5.015 to -0.048	0.077
**C12**	0.001	0.000 to 16.467	0.169	-0.775	4.868	-16.596 to 0.076	0.148
**C14**	0.000	0.000 to 61227	0.176	-2.909	22.167	-70.102 to 6.634	0.213
**C14:1**	1.272	0.003 to 530.3	0.938	-0.695	4.288	-9.355 to 5.900	0.949
**C16**	0.000	0.000 to 95.278	0.178	-0.407	7.400	-24.807 to 3.196	0.150
**C16 OH**	0.000	0.000 to28000	0.172	-8.921	183.7	-634 to 56.1	0.148
**C18**	0.000	0.000 to 90242	0.191	-1.559	17.136	-59.359 to 5.353	0.156
**C18:1OH**	0.000	0.000 to 64370	0.381	-4.776	82.936	-264.4 to 72.25	0.333
**C18:1**	0.400	0.000 to 620	0.807	-0.219	3.482	-8.050 to 4.983	0.779
**C2/C3**	0.956	0.910 to 1.004	0.073	-0.003	0.024	-0.100 to -0.011	0.040
**C8/C10**	0.010	0.000 to 1.895	0.085	-0.231	2.921	-11.03 to -0.034	0.113
**C2/C10**	0.961	0.924 to 0.999	0.042	-0.003	0.024	-0.091 to -0.005	0.070
**C2/C14:1**	0.993	0.986 to 1.001	0.086	-0.001	0.005	-0.018 to 0.000	0.127
**C0/(C16+C18)**	0.994	0.988 to 1.000	0.071	0.000	0.003	-0.012 to -0.002	0.028
**C2/(C16+C18:1)**	0.942	0.908 to 0.977	0.001	-0.001	0.019	-0.100 to -0.026	0.001

Table 4 shows the results from forward stepwise multivariable Cox regression analysis.

**Table 4:** Multivariable Cox regression analysis for the correlates of mortality by forward stepwise method and bootstrap results.

	Hazzard ratio: Exp(β)	95%CI	*P*	Bootstrap Results (2000 Replicas)			
				Bias	S.E	95% CI	*P*
**diabetes states(No = 0, Yes = 1)**	4.981	2.107 to 11.77	<0.001	0.041	0.493	0.719 to 2.778	<0.001
**Age (per 1 year)**	1.052	1.014 to 1.091	0.006	0.002	0.025	0.006 to 0.110	0.023
**C2/(C16+C18:1) (per 1)**	0.937	0.904 to 0.971	<0.001	-0.001	0.016	-0.096 to -0.037	<0.001

footnote: C2,Acetylcarnitine; C16, Palmitoylcarnitine; C18:1 Octadecenoylcarnitine.

The diabetes state, age, and C2/(C16+C18:1) level were determined to be significant prognostic factors. The diabetic state and age showed a positive correlation, while the C2/C16+C18:1 level showed a negative correlation to the survival rate. These factors were also selected using the backward stepwise method. To verify the reliability of the selected factors, bootstrapping multivariable Cox regression analysis of the diabetes state, age, and C2/(C16+C18:1) level was conducted through the brute force approach. The results from this analysis also showed that both the factors were significant.

We divided the patients according to the median 41 of C2/(C16+C18:1) ratio into high group and low group and compared the survival rates between these groups. Table 5 shows the clinical characteristics of the two groups. No significant differences were observed between age and diabetes states selected in the multivariable Cox regression analysis.

**Table 5:** Clinical characteristics of the high and low groups.

Variable	High group (N = 58)	Low group (N = 53)	Mann-Whitney's U test P value	Bootstrap Results (2000 Replicas) P value
**Age (year)**	57.8±14.8 59 [47, 68]	61.1±13.7 61 [53, 71]	0.318	0.228
**Gender (Male/Female)**	39/19	31/22	0.431	
**Etiology of ESRD** **(DM/ not DM)**	14/44	7/46	0.155	
**Dialysis vintage (year)**	10.6 ± 10.7 7 [2, 15]	13.4±12.0 9[2,24]	0.293	0.200
**Body Mass Index (kg/m2)**	20.7±4.1 19.9 [17.9, 22.0]	20.2±4.2 19.2 [17.4, 22.2]	0.460	0.531
**Hemoglobin (g/dL)**	11.0 ± 1.0, 11.1 [10.5, 11.9]	10.8±1.1 10.8 [10.1, 11.5]	0.130	0.067
**Total protein (g/dL)**	6.7 ± 0.5, 6.7 [6.4, 7.0]	6.7±0.4 6.7 [6.5, 7.0]	0.683	0.730
**Albumin (g/dL)**	3.7 ± 0.4, 3.7 [3.4, 3.9]	3.6±0.4 3.7 [3.5, 3.8]	0.794	0.486
**Transferrin saturation: TSAT (%)**	22.1 ± 10.8, 21.0 [12.8, 29.2]	19.3±9.1 17.4 [12.2, 26.3]	0.184	0.145
**Ferritin (ng/mL)**	55.9 ± 66.2, 33 [18, 71]	59.4±47.5, 44 [22, 96]	0.173	0.749
**C-reactive protein (CRP) (mg/dL)**	0.44 ± 1.09, 0.11 [0.06, 0.27]	0.51±0.93, 0.12 [0.04, 0.41]	0.873	0.705
**spKt/V un**	1.46 ± 0.33, 1.41 [1.28, 1.62]	1.54±0.28, 1.51 [1.32, 1.73]	0.110	0.187
**Whole parathyroid hormone(wPTH) (pg/mL)**	137 ± 111, 111 [58, 176]	128±120, 96 [50, 145]	0.381	0.668
**β2-microglobulin (mg/L)**	28.1 ± 6.6, 27.5 [24.7, 32.7]	28.0±5.4, 28.5 [23.8, 31.5]	0.925	0.934
**Erythropoiesis-stimulating agent (ESA) doses/kg/(g/dL)/week**	11.0 ± 12.0, 9.1 [3.1, 14.3]	12.7±13.1, 10.3 [4.5, 13.0]	0.276	0.483
**Pre-dialysis systolic blood pressure (mmHg)**	140 ± 22, 139 [125, 154]	145±24, 144 [132, 161]	0.275	0.273
**Pre-dialysis diastolic blood pressure (mmHg)**	79 ± 17, 81 [73,86]	78±16 78 [69, 89]	0.620	0.856

**Fig 1:**
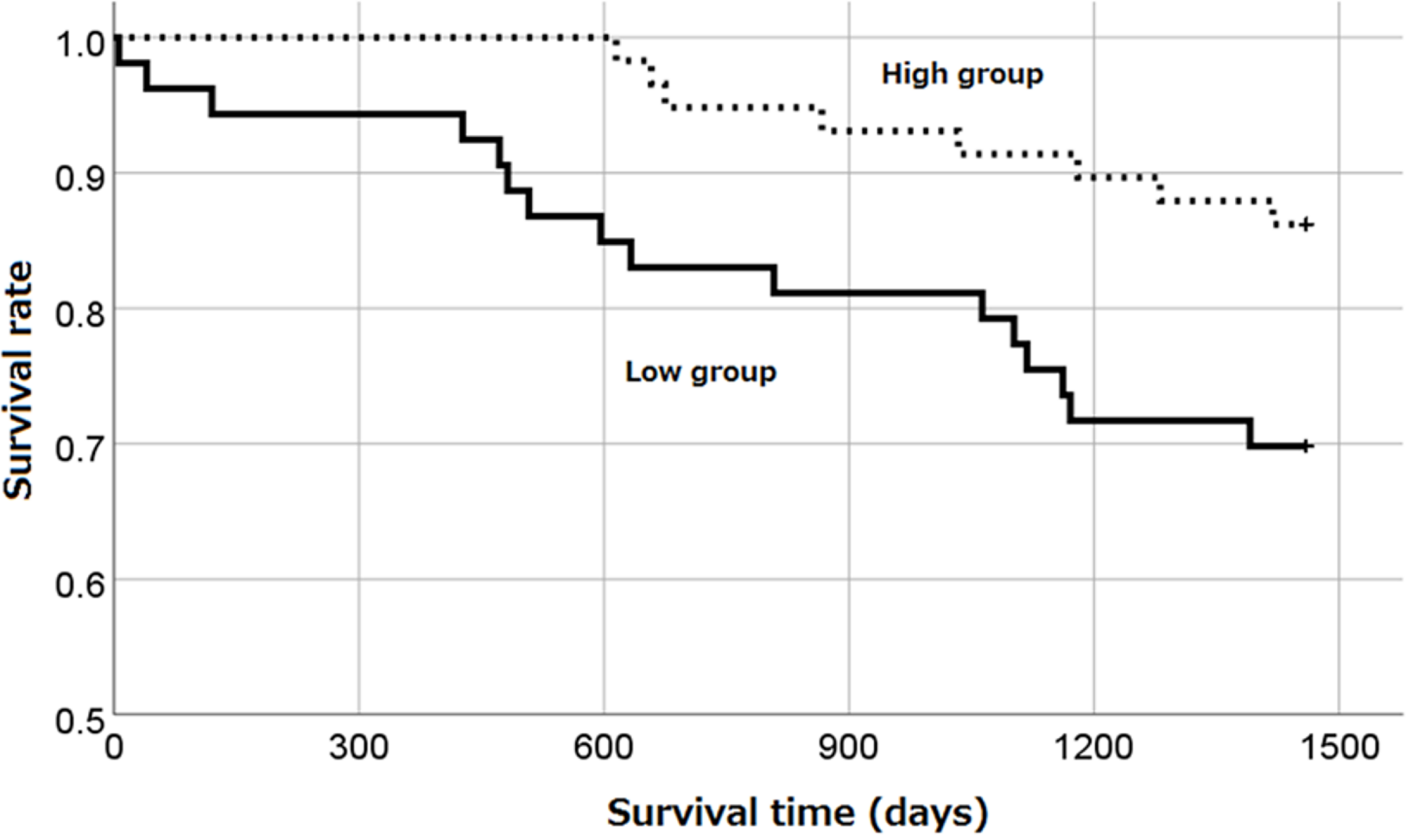
The survival probabilities of hemodialysis patients in high C2/(C16+C18:1) group or low C2/(C16+C18:1) group (log-rank test, P = 0.027. Breslow test, P = 0.021).

Fig 1 shows the survival rates of these two groups as analyzed by Kaplan–Meier analysis, log-rank test, and Breslow test. The observation period was for 4 years, and the survival rates were 86.2% and 69.7% in the high and low groups, respectively. The survival rate was significantly higher in the high group than that in the low group (log-rank test; p = 0.027, Breslow test; p = 0.021).

## Discussion

The present study revealed that 4-year all-cause mortality is positively correlated with the age and diabetes state and negatively correlated with the C2/(C16+C18:1) ratio.

We made a novel discovery of correlation between C2/(C16+C18:1), which represents the fatty acid β-oxidation efficiency, and the mortality rate. It has been reported that, among hemodialysis patients, those with diabetes possess a significantly lower survival rate than those without diabetes [16]. In addition, our finding that age and diabetes are related to 4-year mortality rate also appears to be reasonable.

Fatty acid oxidation is the primary metabolic pathway in diverse tissues, which becomes particularly important during the periods of glucose deprivation. In organs such as the liver and skeletal muscle, fatty acid oxidation can provide >75% of cellular ATP, while in the cardiac tissue, it can be responsible for up to 90% of cellular energy requirements [17,18].

Impaired β-oxidation of fatty acids causes intracellular accumulation of toxic lipids, which in turn initiates a vicious cycle that leads to further cellular dysfunction and death, thereby contributing to CKD, irrespective of the underlying etiology [19].

The C2/(C16+C18:1) ratio is used as an index for diagnosing carnitine palmitoyltransferase-2 (CPT2) deficiency (OMIM 600650) and carnitine-acylcarnitine translocase (CACT) deficiency (OMIM 212138).

In CPT II and CACT deficiencies, the carnitine pathway enzymes are impaired, thereby making it impossible for long-chain fatty acids to be absorbed into the mitochondria, which further impair the β-oxidation of long-chain fatty acids. This event leads to an increase in the long-chain acylcarnitine (e.g., C16, C18, and C18:1), with no elevation in acetylcarnitine (C2), resulting in a high C16+C18:1/C2 ratio.

Carnitine deficiency in hemodialysis patients is believed to impair the uptake of long-chain fatty acids into the mitochondria, resulting in β-oxidation dysfunction. ATP is essential for the biological activity, and β-oxidation efficiency is considered to be an important factor in relation to prognosis. In this study, the C2/(C16+C18:1) ratio, which represents the β-oxidation efficiency, was selected as a factor that is correlated with prognosis of the aforementioned reasons.

Sepsis-related multiple organ failure (MOF) is characterized by immune, endocrine, metabolic, and circulatory failure due to hyperinflammation. Mitochondrial dysfunction plays an important role in the mechanism of MOF [20]. In severe infectious disease such as sepsis, glucose is first recruited and used for energy metabolism in the acute phase, and in the later phase of infection, fatty acid metabolism—such as ketone body production and fatty acid β-oxidation—becomes predominant [21–25].

Pyruvic acid, the end product of glycolysis, converts lactic acid or is utilized by the TCA cycle as fuel. Pyruvate dehydrogenase, which converts pyruvate to acetyl-CoA, becomes less active in cases of sepsis [26]. However, previous studies have reported that ATP concentrations in patients with sepsis are not significantly different from those in healthy individuals [27].

Experiments using an animal sepsis model and clinical studies in sepsis patients studying changes in lipid metabolism and metabolome analysis between survivors and non-survivors suggest a decrease in the fatty acid β-oxidation ability and accumulation of long-chain acylcarnitine [28–30], and some studies have examined the effects of carnitine supplementation treatment [31–33].

This switching of energy sources is very complicated and differs in the liver, adipose tissue, kidney, diaphragm, and heart. In whole body energy metabolism, liver fatty acid metabolism is important for recovery from severe infections such as sepsis [31, 34–35].

The switching of energy metabolism is regulated by the expression of fatty acid β-oxidation enzymes, the carnitine system, and PPARα,β/δandγ; these systems are downregulated and show complicated changes in response to inflammatory cytokines, such as TNFα and lipopolysaccharide, released in severe infectious diseases [29,36,37].

It has been well established that carnitine plays a very important role in β-oxidation, free carnitine is greatly reduced, and acylcarnitine—especially long chain acylcarnitine—accumulates in dialysis patients.

Although C2/(C16+C18:1), which is thought to indicate fatty acid β-oxidation efficiency, significantly correlates with prognosis, the above factors are considered as additional possible reasons for its use.

The heart muscles require a large amount of energy, and most ATP production is dependent on the β-oxidation of long-chain fatty acids. Heart failure is a major cause of death among hemodialysis patients.

Free carnitine is decreased and acylcarnitine is increased in the so-called carnitine deficiency state in dialysis patients [2, 38].

Several studies have reported changes in the plasma concentrations of free carnitine, total carnitine, and acylcarnitine; however, only few studies have reported acylcarnitine profiles or molecular species in dialysis patients. Thus, it is important to study acylcarnitine profiles. Recently, it has been reported that long-chain acylcarnitine, such as palmitoylcarnitine, serves as an excellent prognosis biomarker for morbidity/mortality in cardiovascular patients [39–42], which is also true for heart failure in dialysis patients [43].

Moreover, L-carnitine administration to hemodialysis patients with cardiac hypertrophy or cardiac hypofunction reportedly improves the ejection fraction, left ventricular mass index, cardiothoracic ratio, and intradialytic hypotension [44–48].

Fatty acid oxidation occurs in the mitochondria and peroxisomes. Peroxisomes preferentially oxidize longer chain fatty acids, whereas mitochondria have higher specificity for shorter chain fatty acids [49]. Unlike in mitochondria, beta oxidation does not depend on L-carnitine in peroxisomes.

When fatty acids are decomposed to octanoyl-CoA, the reaction stops. Peroxisomes have a broader substrate selectivity than mitochondria [50].

Fatty acid oxidation in peroxisomes is important for regulation and special functions. Dysfunction in fatty acid oxidation may be related to the division of labor between peroxisomes and mitochondria. Therefore, the activation of β-oxidation in mitochondria and peroxisomes may improve long-chain fatty acid metabolism and vital prognosis.

If β-oxidation improves with L-carnitine administration, it is possible that L-carnitine administration improves the disease prognosis. Therefore, the C2/C16+C18:1 ratio may be a potential indicator for the efficiency of L-carnitine administration in the longevity of hemodialysis patient.

Although the precision of tandem mass spectrometry is unquestionable, we believe that slight differences arise in the measurements when it is performed across different facilities or reagent batches. Therefore, quality control is important, as demonstrate by a previous study that reported differences of approximately 1.6-fold resulting from the use of different measurement methods for serum ferritin [51]. In our study, the C2/C16+18:1 ratio cutoff value was set at 41 (median). However, this value was limited to our study alone. If L-carnitine administration, which can improve the long-chain fatty acid metabolism, could lead to improved prognosis; in the future, the reference values for L-carnitine administration should be investigated with due consideration to the medical expenditure.

### Limitation

The present study had several limitations. First, we could not exclude the potential effect of other unknown confounders. Second, our study was a observational design based on a single-center cohort. Although we confirmed the reliability of explanatory variables selected by stepwise analysis using bootstrap method, further investigation including a larger sample size from multiple centers is necessary for external validity. Finally, it is necessary to ascertain whether the association observed between the carnitine profile and all-cause mortality in the present study was a correlation or a causal relationship. A large-scale randomized controlled study should be conducted to verify the association between all-cause mortality and changes in the carnitine profile due to L-carnitine administration.

## Conclusion

The 4-year all-cause mortality negatively correlated with the C2/(C16+C18:1) ratio. Improvement of impaired β-oxidation state may thus ameliorate prognosis. However, further investigation is necessary to ascertain our findings.
